# Nirsevimab prophylaxis on pediatric intensive care hospitalization for severe acute bronchiolitis: a clinical and economic analysis

**DOI:** 10.1186/s13613-025-01460-0

**Published:** 2025-04-26

**Authors:** Sarah Touati, Alexandre Debs, Luc Morin, Laure Jule, Caroline Claude, Pierre Tissieres, the PANDORA study group, Sarah Touati, Alexandre Debs, Luc Morin, Laure Jule, Caroline Claude, Melissa Beggaz, Matthieu Blanc, Claire Boithias, Charlotte Brognion, Diane Carriere, Ramy Charbel, Mafoudia Conde, Vladimir L Cousin, Narjess Ghali, Clémence Marais, Jordi Miatello, Francisco Oddone, Pierre Tissières

**Affiliations:** 1 IHU-PROMETHEUS Comprehensive Sepsis Center and Pediatric Intensive Care, Neonatal Medicine, and Pediatric Emergency Department, AP-HP Paris Saclay University, Bicêtre Hospital, 78 Rue du Général Leclerc, 94275 Le Kremlin-Bicêtre, France; 2 Institute of Integrative Biology of the Cell, CNRS, CEA, Paris Saclay University, Gif-Sur-Yvette, France

**Keywords:** Respiratory syncytial virus, Bronchiolitis, Intensive care, Nirsevimab, Cost-effectiveness

## Abstract

**Background:**

Severe acute viral bronchiolitis is a common cause of admissions to pediatric intensive care units (PICUs), resulting in a significant organizational burden each winter. The recent introduction of generalized neonatal prophylactic therapies using Nirsevimab, a monoclonal antibody targeting the respiratory syncytial virus (RSV), has significantly reduced consultations and hospitalizations. However, its impact on the medico-economic aspects of the PICU remains poorly defined.

**Methods:**

We analyzed all infants admitted to our unit for severe acute bronchiolitis over six consecutive epidemic periods (September to March) and examined the effect of Nirsevimab generalized prophylaxis on PICU admissions during the 2023–2024 period.

**Results:**

Between 2018 and 2024, 572 out of, 3728 infants under 6 months of age were admitted to the PICU for severe acute bronchiolitis during six epidemic periods. The average percentage of infants with bronchiolitis admitted to the PICU was 15.3% (95% CI 14.2–16.5), with a net decrease during the 2023–2024 period (8.5%) compared to the 2022–2023 period (17.6%). Patients’ characteristics were similar, as were the supporting therapies. The causes of bronchiolitis were identical, with 83% and 77% secondary to RSV. PICU duration was significantly reduced during the last period from 4.4 days (95% CI 3.8–5.9) to 3.3 days (95% CI 2.6–4). The medico-economic impact was significant, with a cost reduction for acute severe viral bronchiolitis PICU total stays from €210,105 to €121,044 per annual epidemic without a change in the return on investment.

**Conclusions:**

The introduction of generalized neonatal prophylaxis with Nirsevimab significantly impacts the burden of severe acute bronchiolitis in the PICU.

## Take-home message

Prophylactic administration of Nirsevimab, An anti-VRS monoclonal antibody, after delivery significantly affects the burden of severe acute viral bronchiolitis in pediatric intensive care

## Introduction

Bronchiolitis is the leading cause of acute viral infection of the lower respiratory tract, affecting infants, and is associated with significant public health concerns [1]. The Respiratory Syncytial Virus (RSV) is responsible for most severe acute bronchiolitis cases and hospitalizations in pediatric intensive care units (PICUs). Even if prevention with Pavilizumab is available for at-risk neonatal populations, healthy infants account for most hospital admissions. Recently, prophylactic administration in newborns of Nirsevimab, a monoclonal antibody targeting RSV, showed a significant reduction in the clinical burden of the RSV annual epidemic [2, 3]. This effect was confirmed by real-life case–control studies [4] encouraging health authorities to implement neonatal prevention in many countries, including France. Consequently, the present report aims to evaluate the effectiveness of Nirsevimab neonatal prophylaxis in reducing the number of PICU hospitalizations for severe acute bronchiolitis, its effect on the severity, and the economic impact in a referral PICU.

## Methods

We analyzed a surveillance database of all infants younger than 6 months of age admitted to the PICU for bronchiolitis across six consecutive winter seasons (from September 2018 to March 2024), encompassing the marketing of Nirsevimab in France (winter 2023–24) and the implementation of barrier measures to limit the spread of SARS-CoV-2 (winter 2021–22). Infants diagnosed with asthma were excluded. The primary outcome was the incidence of bronchiolitis PICU hospitalization; the secondary outcome was the incidence of RSV infection, the severity of the disease, and its economic impact. Demographic characteristics were prospectively obtained, including the duration and mode of ventilator support, use of antibiotics, and infection-related markers such as procalcitonin, microbiological cultures (blood and urine), and respiratory nasopharyngeal sampling for pathogen molecular detection (including respiratory viruses and pertussis). A medico-economic analysis was performed using analytic accounting. Total costs were assessed using the institutional billing database. The cost information was obtained from the hospital’s accounting reports. Costs were given per day of hospitalization in the unit. This global accounting cost includes all associated costs, staff salaries, equipment, procedures, and amortization of premises. This value is calculated for each hospital unit based on actual expenses paid in the previous year. The billing costs represent the effective payments obtained from the health care system. They were calculated based on national accounting for disease diagnosis and severity per day of hospitalization in the PICU. The cost burden for both periods was estimated using the median length of stay (LOS) in the PICU. We used the analytic cost per stay (mean values of cost from 2018 to 2024 was €1865, 95% CI €1829–€1900) using the following formula: total cost (Euros) = [mean PICU LOS (days) × PICU cost accounting]. Values were expressed as percentage, mean, and 95% confidence interval (CI). Statistical analysis was conducted using R and RStudio [5], using the T-test for continuous data, the Chi-squared test, and Fisher’s exact test for categorical data. A p value was considered as significant when < 0.05.

## Results

**Fig. 1:**
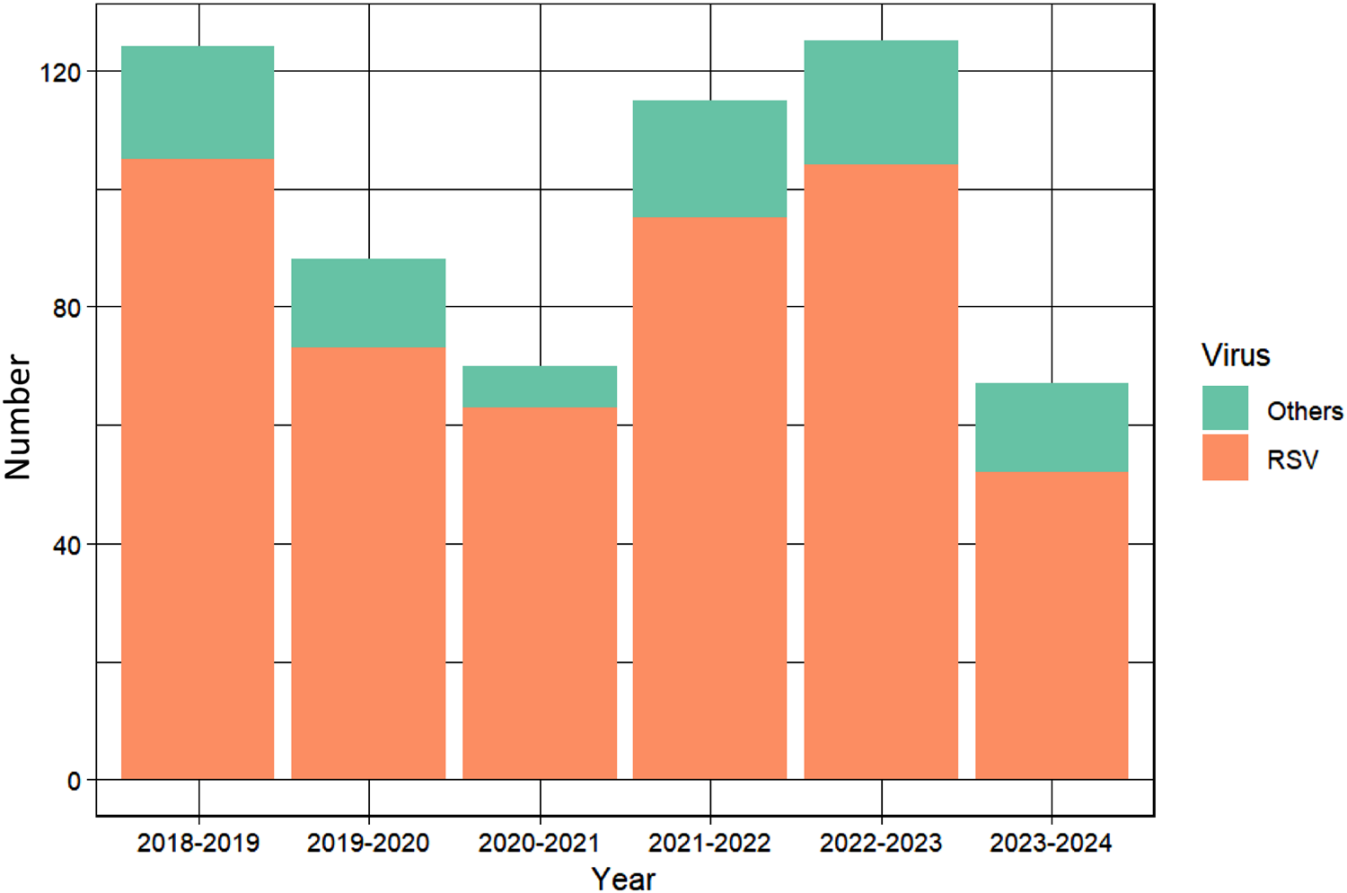
Incidence of severe acute viral bronchiolitis from 2018 to 2024. The incidence of Respiratory Syncytial Virus and other causes of severe acute viral bronchiolitis in children admitted in intensive care. Values are expressed as number

During the study period, 572 (15.3%) out of 3,728 children under 6 months of age with severe acute viral bronchiolitis were hospitalized in the PICU. During the 2023–2024 winter epidemic, 772 patients under 6 months were hospitalized in the PICU for all morbidities combined and 67 (8.7%) for severe acute bronchiolitis, of whom 21 received prophylaxis with Nirsevimab before admission. Comparing the incidence of hospitalization for bronchiolitis during the winters before (2022–23) and following the Nirsevimab post-marketing period (2023–24) (Fig. 1), our study revealed a significant decrease in the total number of hospitalizations for all viral causes of bronchiolitis, namely 17.6% (95% CI 14.9–20.7) versus 8.5% (95% CI 6.8–10.7, *p* < *0.001*) (Table 1). There was a concomitant decrease in the duration of PICU stays (4.4 days [95% CI 3.8–5.1] vs. 3.2 days [95% CI 2.7–3.4], *p* = *0.02*). Patient characteristics were similar between the two periods. The age at admission and the rate of former prematurity were not statistically significant between the two periods. The rate of RSV infection among bronchiolitis in PICU patients remained unchanged between the two periods (83% vs. 77%, *p* = *0.4*). The rates of invasive and non-invasive ventilation and the use of antibiotics were similar between the two periods. The number of severe acute bronchiolitis hospitalizations during the Nirsevimab post-marketing period (winter 2023–24) was identical to that observed in winter 2020–2021, a period characterized by the implementation of barrier measures to limit the spread of SARS-CoV-2. In 2023–2024, among the 21 patients who received Nirsevimab before their hospitalization, RSV was positive in 33% compared to 84.9% in untreated patients (61.9% reduction, *p* < *0.001*). The costs used in this study, obtained from the hospital accounting reports, were an average of €351 (95% CI 327–374) per day of PICU care (Table 2). We did not measure the individual costs. On an annual basis, hospital costs remained stable between the last two periods at €1827 and €1834 per PICU stay. Along with reducing the length of the PICU, we calculated a total cost reduction during the 2023–2024 period of €89,061 per annual epidemic period. The return on investment remains stable at 89.9% and 89.1%, respectively, outlining a minimal effect on the cost-effectiveness of Nirsevimab prophylaxis on hospital accounting despite a significant impact on the acute severe bronchiolitis PICU burden.

**Table 1:** Patients characteristics

	2018–2024	2022–2023*	2023–2024*	*p* value*
Patients with bronchiolitis/total (number)	572/3728	115/654	66/772	< *0.001*
Demographics				
< 3 months old (%)	80% (77–84)	81% (74%–88%)	73% (62%–84%)	*0.28*
Age at admission (days)	54.1 (50.5–57.7)	53.9 (45.9–61.9)	65.3 (53.1–77.5)	*0.12*
Gestationnal age (weeks)	36.9 (36.5–37.4)	36.6 (35.7–37.5)	37.5 (36.6–38.5)	*0.15*
Viral causes^†^				
Respiratory syncytial virus (%)	84% (81–87)	83%	77%	*0.40*
Rhinovirus (%)	15% (12–18)	14%	23%	*0.19*
CPAP (%)	92% (90–94)	93%	94%	*1*
Bi-PAP (%)	10% (8–13)	15%	14%	*1*
High flow oxygenation (%)	10% (8–13)	1%	3%	*0.6*
Invasive ventilation (%)	5% (3–7)	5%	5%	*1*
Antibiotherapy (%)	21% (18–24)	22%	24%	*0.9*
Outcome				
Lengh of stay in PICU (days)	4.15 (3.8–4.5)	4.42 (3.8–5.1)	3.32 (2.7–4)	*0.02*
Hospital death (number)	1	1	0	*1*

Values are expressed as mean or percent (95% confidence interval, if relevant)

*CPAP* continuous positive airway pressure, *Bi-PAP* biphasic positive airway pressure, *PICU* pediatric intensive care unit

^*^Comparison between 2022–2023 and 2023–2024 epidemic seasons

^†^Patients may have respiratory pathogens detected by PCR such Influenza virus, adenovirus, SARS-CoV2, other Coronavirus, Metapneumovirus, *Bordetella pertussis*, and Parainfluenzae virus

**Table 2:** Analytic Accounting

	2018–2024	2022–2023	2023–2024
Cost (Euro)	177,974 (128,458–227,489)	210,105	121,044
Billing (Euro)	331,210 (239,936–422,485)	398,935	228,954
Return on investment (%) ^‡^	86.9% (83.4–90.3)	89.9%	89.1%

Values are expressed as the mean (95% Confidence Interval)

^‡^ Return on investment corresponds to the following calculation: (Billing − Cost)/Cost × 100

## Discussion

Our findings indicate a notable reduction in hospitalizations and PICU length of stay during the winter of 2023–2024 compared to 2022–2023, suggesting an effect of Nirsevimab generalized prophylaxis on the severe acute bronchiolitis PICU burden.

The impact of public health measures to prevent the dissemination of infectious diseases has been shown to be highly effective in minimizing pediatric intensive care admissions. From pertussis to Haemophilus influenzae type b, pneumococcal, and meningococcal vaccinations, all have shown a dramatic decrease in the burden of severe infections and associated mortality. Targeting the respiratory syncytial virus is a further step toward minimizing infant infectious disease burden. Acute viral bronchiolitis significantly impacts the healthcare system’s organization. Access to prophylactic measures to prevent the development of acute viral bronchiolitis is highly anticipated. Although monoclonal antibodies targeting RSV have shown utility in high-risk neonates, dissemination to a larger population has been limited. A new generation of monoclonal antibody against RSV, Nirsevimab, has demonstrated its efficacy in preventing RSV bronchiolitis in high-risk premature and term infants. The ease of administration and safety allowed for the demonstration in the Harmonie Study of its effectiveness in decreasing the occurrence of RSV bronchiolitis when administered prophylactically as early as at birth [2]. After implementing neonatal prophylactic strategies using Nirsevimab in France starting in 2023, the impact on the burden of the healthcare system was assessed.

In this study, we demonstrated a significant decrease in both the number of PICU admissions and the length of stay following the introduction of neonatal prophylaxis. The net effect was substantial, with a reduction from 17.6 to 8.5% (− 9%) in overall hospitalizations for children under 6 months of age during the epidemic period and a decrease from 4.4 to 3.2 days (− 25%). This exceeds the expected reduction in all U.S. PICU encounters and ICU days, which were reductions of 2.1–2.8% and 4.5%–5.9%, respectively [6]. Alejandre et al. in a similarly designed study, confirmed the net effect of Nirsevimab neonatal prophylaxis in reducing PICU hospitalizations of infants with bronchiolitis by 50%, but did not demonstrate a reduction in the duration of PICU stay. Potential variability in management, including more mechanical ventilation than seen in our study, was also noted, along with a higher prevalence of co-morbidities. Interestingly, the demographics and severity of patients remained unchanged between periods.

The crude efficacy for all admitted infants (irrespective of the viral cause) was 60.3%, and in RSV-positive bronchiolitis, it was 72.2% (respectively 14% and 86.2% during the 2022–2023 and 2023–2024 period). The efficacy was below that seen in similar studies from the Madrid region and Catalonia, where crude efficacy in RSV infants varied between 75 and 99% [7, 8]. In a case–control study from 20 PICUs in France, effectiveness was estimated to be 74.4% [9].

In our study, among admitted infants with bronchiolitis (including RSV negative), 21 out of 66 infants (32%) had received Nirsevimab; 33% (7/21) and 97.8% (44/45) were RSV positive, respectively. In contrast, Alejandre et al*.* showed, during the 2023–2024 epidemic period, a higher proportion of immunized infants (35/52, 67%) with an identical proportion than in our study being RSV positive (12/35, 34%) [10]. This highlights the necessity of considering the potential lack of efficacy in certain infants (e.g., the development of Nirsevimab-resistant RSV strains and patient-related risk factors) and the gap in the optimal prevention strategy. In our cohort, all Nirsevimab injections were administered during the first few days after birth before returning home. Delays in providing immunization may affect the optimal protection of infants, and a combined approach involving maternal vaccination could help bridge the existing gap.

Modifying the management of acute viral bronchiolitis has been shown to significantly impact the clinical and economic burden. Essouri et al*.* demonstrated in a seminal paper that changing the mode of ventilatory support for bronchiolitis admitted to the PICU strongly affects the duration of hospitalization and economic outcomes burden [11]. Here, using a prophylactic approach to RSV bronchiolitis we further confirms that a global change in bronchiolitis management, even before the disease occurs, significantly impacts the PICU burden. The observed net effect of Nirsevimab prophylaxis on PICU admissions is even strengthened, as it was similar to the barrier measures implemented to limit the spread of SARS-CoV-2 during the 2021–2022 epidemic period. This confirms Alejandre et al*.*’s report showing a similar bronchiolitis PICU encounters between the 2021–2022 and 2023–2024 periods [10].

Establishing the cost-effectiveness of such a population-based prophylactic strategy is essential. Hutton et al*.* developed a decision analytical model using secondary data simulation, such as data obtained from published literature, Food and Drug Administration approval documents, and epidemiologic surveillance data. They estimated that 14,341 hospitalizations could be averted each year if half of the US birth cohort receives Nirsevimab, with an estimated cost-saving per quality-adjusted life year (QALY) of $323,788. They concluded that neonatal Nirsevimab prevention may be cost-effective among those with higher risks [12]. This is also suggested by a meta-analysis evaluating 28 economic evaluations, showing that Pavilizumab as an RSV prophylaxis is cost-effective for high-risk patients, such as premature infants, infants with chronic pulmonary disease, and infants from remote communities. However, the range of the cost-effectiveness ratio (per QALY) was elevated and subject to important interfering factors [13]. Similarly, the cost-effectiveness of administering Nirsevimab as neonatal prophylaxis is sensitive to various factors. The price per dose (PPD) of Nirsevimab could be highly influential in determining whether Nirsevimab is cost-effective. RSV-associated healthcare costs may vary according to severity, with PICU hospitalization carrying the heaviest economic burden per capita. Our data provide essential information on the impact of Nirsevimab neonatal prophylaxis on cost-effectiveness in the French healthcare system, showing a significant effect on the duration and absolute number of PICU hospitalizations without impacting the return on investment from a microeconomic perspective. The uncertainty of the global cost-effectiveness of administering Nirsevimab at birth is a further challenge with the possibility of a vaccine for the mother. Shoukat et al*.* recently analyzed the cost-effectiveness of Nirsevimab neonatal prophylaxis and maternal RSVpreF vaccination in Canada. Interestingly, based on a willingness-to-pay model of CAD $50,000 per QALY gained, the PPD of Nirsevimab would be $290, corresponding to a budget of $83,978 for 1113 infants per 100,000 population [14]. In France, the negotiated cost of Nirsevimab is 401,80 Euros per dose, with a target to cover all births. The cost-effectiveness of this prophylactic population-based strategy has not been addressed, but the current PPD and its impact need to be evaluated.

This study has several limitations. First, the single-center design may not be representative, as management strategies may directly impact the PICU stay and associated morbidities and mortality [11, 15, 16]. Second, the impact of neonatal prophylaxis on the clinical and economic burden may vary widely according to population coverage. Only a national cost-effectiveness study could provide more robust results. Implementing the effects of maternal vaccination is indeed necessary. In our cohort, no mothers received RSVpreF vaccination. Third, patients’ stratification regarding their socio-economic profile may be necessary. Studies have identified high-risk RSV infants among specific communities where access to healthcare is constrained or genetic susceptibility to respiratory infections is established. A population-based analysis is essential to better identify high-risk populations and their relative effects on QALY.

In conclusion, this study shows that Nirsevimab prophylaxis at birth significantly reduces the PICU burden by decreasing the number and length of stays in the PICU. In addition, a medico-economic analysis suggests that this decrease in the PICU burden does not affect the return on investment for infants admitted for severe acute bronchiolitis. The net benefit of Nirsevimab prophylaxis on the epidemic management of acute severe bronchiolitis seems positive. Still, careful attention to the evolution of RSV-related bronchiolitis in Nirsevimab-treated infants, monitoring the development of potential RSV strain resistance, and identifying risk factors are necessary.

## Data Availability

Data will be available to the contact author upon reasonable request.

## Abbreviations

RSV: Respiratory syncytial virus
PICU: Pediatric intensive care unit
CI: Confidence interval
